# The valid publication of *Salix suchowensis* (Salicaceae)

**DOI:** 10.3897/phytokeys.131.37065

**Published:** 2019-09-02

**Authors:** Li He, Shuai Liao, Wendy Applequist, Shipin Chen

**Affiliations:** 1 College of Forestry, Fujian Agriculture and Forestry University, Fuzhou 350002, China Fujian Agriculture and Forestry University Fuzhou China; 2 School of Life Sciences, East China Normal University, Shanghai 200241, China East China Normal University Shanghai China; 3 Missouri Botanical Garden, St. Louis, MO 63110, USA Missouri Botanical Garden St. Louis United States of America

**Keywords:** gathering, lectotypification, nomenclature, Salicaceae, *Salix suchowensis*, type citation, validation

## Abstract

The nomenclatural problems of *Salix suchowensis* have been addressed by different authors with varying opinions. However, these efforts were flawed by a lack of observation of relevant specimens. Accordingly, we carefully checked relevant publications and specimens both through internet databases and herbarium visits. Here, we thoroughly review the nomenclatural history of *Salix suchowensis* in light of the new definition of a gathering in the *Shenzhen Code*. We conclude that this name was validly published in the original publication in 1963. Furthermore, a lectotype is designated for the precise application of the name. We hope this article will offer guidance for interpreting similar cases.

## Introduction

*Salix* L. (Salicaceae) is variably estimated to include 350–520 species, which are widely distributed in temperate and boreal regions of the Northern Hemisphere with a few species in Africa and South America (Fang et al. 1999; Brummitt 2007; Argus et al. 2010). There are 275 species and 82 varieties in China, of which 189 species and 74 varieties are endemic (Fang et al. 1999). Taxa of *Salix* are dioecious and have different times of development for flowers and leaves (Skvortsov 1999) which makes their identification very difficult.

*Salix suchowensis* W.C. Cheng (in Cheng et al. 1963: 4) is a riparian shrub willow, which is the first species of the genus *Salix* with full genome sequence and a potential bioenergy crop (Dai et al. 2014). Cheng et al. (1963) described *Salix suchowensis* W.C. Cheng and cited two collection numbers, *C.T. Yang 20640* (♂) and “*C.T. Yang 20641* (♀)”, as type. Both Zhu (1998) and Yu et al. (2011) therefore considered that the name *S. suchowensis* was not validly published by Cheng et al. (1963). In the most recent complete treatment of Chinese *Salix* species (Fang et al. 1999), the species is recognised as *S. suchowensis* W.C. Cheng ex G. Zhu. However, as emphasised by the new definition of “gathering” in the *Shenzhen Code* (Art. 8.2 footnote, Art. 8 Note 1 and Ex. 4; Turland et al. 2018), *C.T. Yang 20640* and “*C.T. Yang 20641*” are properly identified as a single gathering. Indeed, the collection number “*C.T. Yang 20641* (♀)” appears to represent a later renumbering of some female duplicates of *C.T. Yang 20640*. Therefore, *S. suchowensis* was validly published with the citation of a single gathering (Art. 40.2 of the *Code*; Turland et al. 2018) and is properly attributed to W.C. Cheng alone. Because the citation of that gathering encompassed multiple duplicates, these are treated as syntypes (Art. 40, Note 1). One of them is herein designated as a lectotype, as recommended by Arts. 9.11–9.12 of the *Code*. Additional details are given below. Since observation of herbarium material has increased the range of variation in some morphological characters beyond that reported by Fang et al. (1999), an updated description and notes on habitat and phenology are provided.

### Historical background and original material

When the name *Salix suchowensis* W.C. Cheng in Cheng et al. (1963: 4) was published, four collections (*C.T. Yang 20640*, *20641*, *10045* and *R.L. Chao 20515*) were cited. Two collection numbers conserved in NF were designated as types with female (“*C.T. Yang 20641*”) and male branches (*C.T. Yang 20640*), respectively.

Zhu (1998) considered that the name was not validly published by Cheng et al. (1963), with two specimens “cited without the indication of a type”, contrary to Articles 8.1 and 37.1 of the *Tokyo Code* (Greuter et al. 1994). To validate the name, Zhu referenced the effectively published Latin description and diagnosis of Cheng et al. (1963) and designated *J.L. Guo 89012* (NF) as the holotype. However, the collection *J.L. Guo 89012* (♀) (see discussion below) was not cited in the publication of Cheng et al. (1963). Moreover, the specimen *J.L. Guo 89012* (GAUF) is amongst the original material cited by Feng and Guo (1990) for “*Populus wenxianica* Z.C. Feng & J.L. Guo”, which was not validly published but was validated by Zhu (1998) who selected *J.F. Liu 88001* (GAUF) as type. Zhu’s citation of locality data for *J.L. Guo 89012* “TYPE: China. Jiangsu: Nanjing, *J.L. Guo 89012*” differs from Feng and Guo’s (1990) citation of locality data as “Gansu: Wenxian, Zhongzhai, 30 May 1989.” *Salix suchowensis* does not occur in Gansu (Fang et al. 1999). In the Chinese part of their paper, Feng and Guo (1990) cited “郭建林88012” at GAUF, rather than 89012 and Zhu (1998) cited *J.L. Guo 88012* from Gansu as being amongst the original material of *Populus wenxianica* Z.C. Feng & J.L. Guo ex G. Zhu. Therefore, it is probable that a typographical error in the English part of Feng and Guo (1990) led to confusion between two distinct specimens of Salicaceae. However, we have been unable to relocate either Guo’s collections at GAUF or NF, so the identity of *J.L. Guo 89012* cannot be confirmed.

Yu et al. (2011) also considered that *Salix suchowensis* was not validly published by Cheng et al. (1963), “with two gatherings indicated as types”. They attributed valid publication to “W.C. Cheng in S.Y. Jin & Y.L. Chen” in *A Catalogue of Type Specimens (Cormophyta) in the Herbaria of China* (Jin 1994: 599). That work listed a single collection, “*20641.* T(♂): NFU”, as the type of *Salix suchowensis* (Jin 1994). The collector name was omitted (apparently by accident) but the reference to *C.T. Yang* is unquestionable. Neither Jin “Jin & Chen” (1994) nor Yu et al. (2011) cited a specific barcode or accession number. The citation is problematic in that, according to Cheng et al. (1963), the collection numbered *20641* contained fragments with female, not male, inflorescences. Despite this, the restriction to a single gathering and herbarium would suffice for valid publication (Art. 40.2 and Art. 40, Note 1). Therefore, Zhu’s (1998) attempt to validate the name using a problematic type created an illegitimate later homonym, the application of which we are not herein attempting to determine.

**Figure 1.**
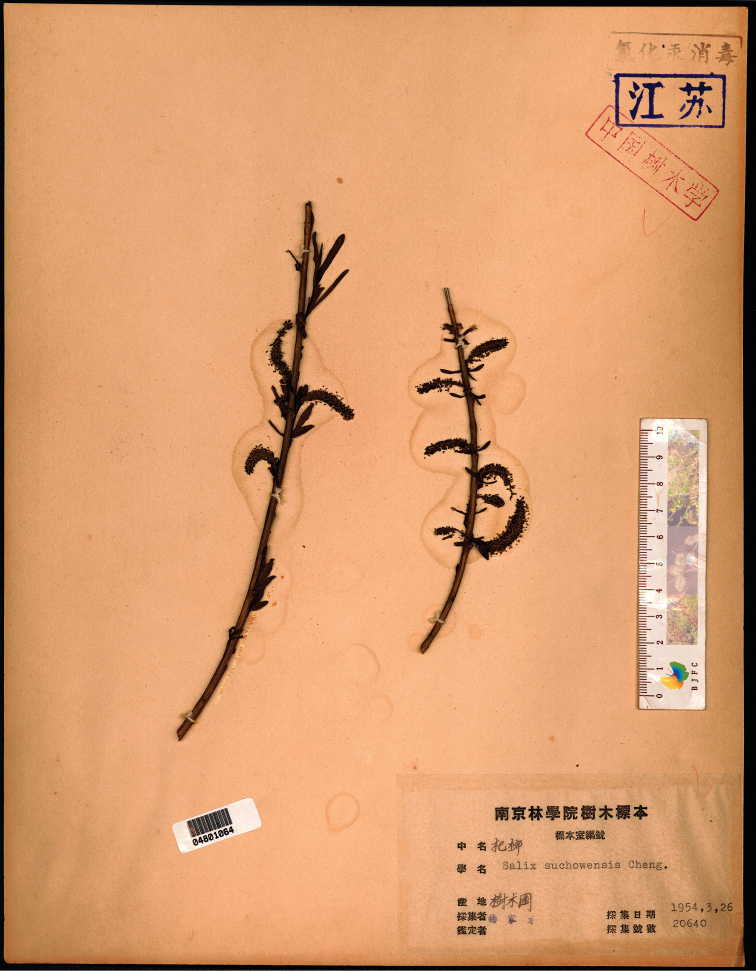
*Salix suchowensis*: *C.T. Yang 20640* (NF barcode 04801064!, ♂, isolectotype).

**Figure 2.**
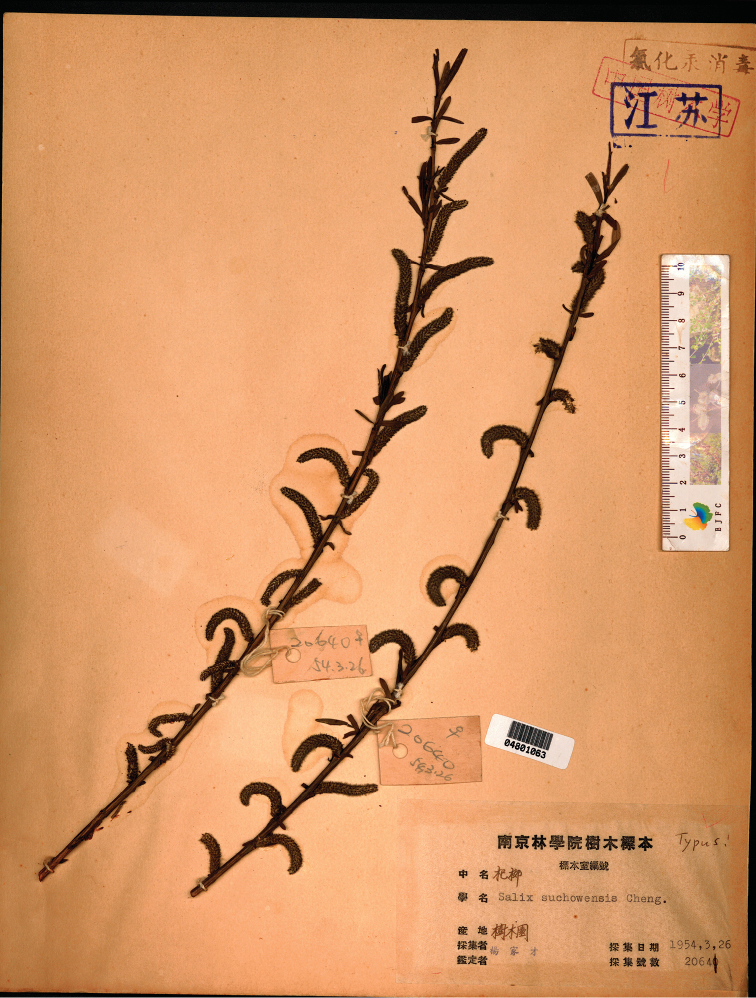
*Salix suchowensis*: *C.T. Yang 20640* (NF barcode 04801063!, ♀, lectotype), renumbered *20641* in herbarium.

After checking all specimens of *Salix* deposited in NF (herbarium acronyms follow Thiers 2019), we found eight duplicates of *C. T. Yang 20640* collected from the Arboretum of Nanjing Forestry University. Seven of them are composed of female branches and one (Fig. 1) is composed of male branches (Table 1). All are dated 26 March 1954. The numbering of two of the female duplicates (NF barcodes 04801051 & 04801063 [Fig. 2], ♀) was later changed to “*20641*” with pen on herbarium labels. However, no duplicates with original numbering of “*C.T. Yang 20641*” were located in NF, nor in other Chinese herbaria (via NSII-National Specimen Information Infrastructure http://www.nsii.org.cn/2017/home.php and CVH-Chinese Virtual Herbarium http://www.cvh.ac.cn/search, both accessed 6 June 2019).

**Table 1.** Original material of *Salix suchowensis* (*C.T. Yang 20640*).

Herbarium	Barcode number	Collection tag	Herbarium label	Sheet contents
NF	04801045	♀, 20640	C.T. Yang 20640	female branches
NF	04801047	♀, 20640	C.T. Yang 20640	female branch
NF	04801048	♀, 20640	C.T. Yang 20640	female branches
NF	04801051	♀, 20640	C.T. Yang 20641 (20640)	female branches
NF	04801060	♀, 20640	C.T. Yang 20640	female branches
NF	04801063	♀, 20640	C.T. Yang 20641 (20640)	female branches
NF	04801064	–	C.T. Yang 20640	male branches
NF	04801068	♀, 20640	C.T. Yang 20640	female branches

The collector C.T. Yang was a colleague of W.C. Cheng who worked a few years for NF then left for the Security Department of Nanjing Forestry University. The location “树木园 [Arboretum]” added by hand to typed herbarium labels is consistent with the handwriting of R.B. Chen, another colleague of W.C. Cheng. This information was obtained from a manager of NF (pers. comm.).

R.B. Chen collected material, later labelled as *Salix suchowensis*, from the Arboretum in 1956 and he participated in the publishing of Cheng’s *Dendrology* [I & II] in 1961 and 1964 (Cheng 1964; Huang 2008). Therefore, we infer that Chen was probably familiar with the process of publication of the name. It is possible that Chen discovered that “*C.T. Yang 20641* (♀)” in the protologue of *S. suchowensis* was labelled *C.T. Yang 20640* (♀) in the herbarium and, while annotating specimen labels, also “corrected” the collection number to match the protologue. The ink used for both annotations is similar. It is not possible to determine with certainty who changed the specimen numbers, nor when. However, the typed labels show that *C.T. Yang 20640* was originally intended by the collector to include both male and female duplicates. This makes it unlikely that Yang himself was responsible for the later renumbering of some (but not all!) of the female duplicates, which, if done by another worker, was inappropriate. Since Art. 9.2 and Ex. 3 of the *Shenzhen Code* permit obvious errors in type citations to be corrected, Cheng’s citation should be corrected to “Kiangsu: *C.T. Yang 20640*, Typus fl. ♂ & ♀!; *C.T. Yang 10045*; *R.L. Chao 20515*”. Under Art. 40, Note 1, all eight specimens of *C.T. Yang 20640* are therefore syntypes of the name.

The “Shenzhen Code” has clarified the definition of a “gathering” as “a collection presumed to be of a single taxon made by the same collector(s) at the same time from a single locality” (Art. 8.2 footnote). Duplicates given different field numbers or collecting numbers, but collected by the same people at the same time and place, can still be treated as a single gathering (Art. 8, Note 1, Ex. 4). Even if the original material of *S. suchowensis* had originally been given two collection numbers (*C.T. Yang 20640*, “*20641*”), as cited by Cheng et al. (1963), or had been renumbered by Yang himself before 1963, they would still properly be treated as part of a single gathering because they belong to a single species and were collected by the same person at the same time from a single locality. Therefore, Cheng would have met the requirements of Art. 40.1 and 40.2 for valid publication of *Salix suchowensis* in Cheng et al. (1963) by the citation of a single gathering as type.

### Typification

Jin’s (1994) faulty listing of “20641. T (♂): NFU” as the type of *Salix suchowensis* does not match any existing duplicate, because neither of the duplicates annotated as “*20641*” are male. Therefore, this listing was insufficient to designate any of the 8 syntype specimens of *C.T. Yang 20640* at NF as lectotype. The same can be said for Yu et al.’s (2011) listing of “*C.T. Yang 20641*” as “holotype”. The attempted designation of type by Zhu (1998) is not allowable because the selected specimen was not amongst the syntypes cited by Cheng et al. (1963), as required by Art. 9.12 of the *Code* (Turland et al. 2018). The sheet with barcode 04801063 is designated here as the lectotype because of its handwritten annotation “Typus” by Chen.

## Taxonomic treatment

### Salix suchowensis

Taxon classificationPlantaeMalpighialesSalicaceae

W.C. Cheng in Cheng et al., Sci. Silvae Sin. 8(1): 4. 1963

B11D84EBCEF35A69B9A977A24593F319

Figures 1 , 2 , 3

#### Lectotype

**(designated here).** CHINA. Jiangsu: Nanjing, Arboretum of Nanjing Forestry University, 26 March 1954, *C.T. Yang 20640* (NF barcode 04801063!, ♀; isolectotypes: NF barcodes 04801045!, 04801047!, 04801048!, 04801051!, 04801060! & 04801068!, ♀, NF barcode 04801064!, ♂). — For image of lectotype, see Fig. 2.

Shrubs. Branches yellowish-green or purplish-red, glabrous; branchlets sparsely tomentose at first, becoming subglabrous. Buds glabrous. Stipules linear to lanceolate, 0.96–1.42(–2.5) cm; petiole 0.3–1 cm, margin remotely glandular dentate, pubescent to glabrous; leaf blade lanceolate, 5.17–12.25 × 0.63–1.7 cm, abaxially pale, both surfaces glabrous, tomentose when young, adaxially dull green, base cuneate, margin glandular denticulate, apex shortly acuminate; lateral veins diverging from midvein at 45–90°. Catkins before leaves emerge, densely flowered. Male catkin terete, 2.2–4 cm × 0.4–0.67 cm, sessile or subsessile, with scale-like leaflets at base; rachis grey tomentose. Female catkin up to 3.43 cm at maturity, sessile or subsessile, with scale-like leaflets at base. Floral bracts long obovate, abaxially villous, apex obtuse-rounded, purple black distally. Male flower: glands adaxial; stamens 2, connate throughout, anthers yellow or reddish-purple. Female flower: ovary conical, densely grey tomentose, ovules 3–7; stipe short to absent; style conspicuous; stigma 2-cleft. Capsule pilose.

**Figure 3.**
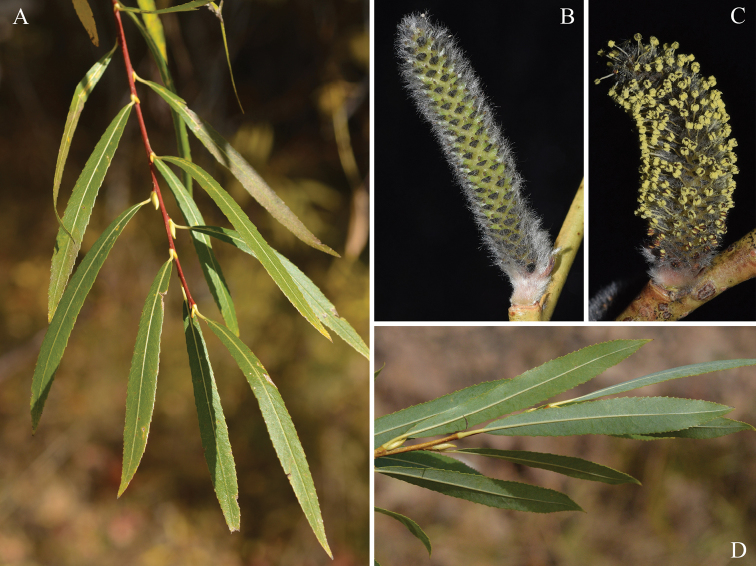
*Salix suchowensis*: **A** branchlet and leaf blades adaxially **B** female catkin **C** male catkin **D** leaf blades abaxially (photo credit: **A, D** by L. He **B, C** by Feiyi Guo).

#### Phenology.

Flowering from March to April and fruiting in April and May.

**Habitat.** Along rivers, stream-sides, or cultivated; near sea level to 900 m a.s.l.

#### Distribution.

Beijing, Hebei, Henan, Jiangsu, Shandong, N Zhejiang.

#### Additional material examined.

—CHINA. **Beijing**: Miyun County, Dajiaoyu, 5 May 1951, *Y. Liu 1507-8* (PE); Yudu Mountain, 900 m a.s.l., 11 June 2019, *F.Y. Guo, Y.M. Wu & D. Liu G2019061105* (BJFC). **Hebei**: Daming County, Dongcao, 100 m a.s.l., 3 June 1972, *Han 165* (PE). **Henan**: Song County, Xiasi, 25 September 1956, *Henanshenglinyeting 1217* (PE). **Jiangsu**: Nanjing, Arboretum of Nanjing Forestry University, 12 April 1954, *C.T. Yang 20645* (NF); ibidem, 26 May 1956, *R.B. Chen s.n.* (NF); Xuanwu Road, 19 May 1956, *C.T. Yang 10045* (NF). **Shandong**: Gudao, Huanghenongchang, 16 July 1959, *T.Y. Zhou 5412* (NAS). **Zhejiang**: Zhuji, Paitou, 24 September 1934, *Y.X. He 2952* (NAS).

## Supplementary Material

XML Treatment for Salix suchowensis
